# Effect of Grain Size on the Corrosion Behavior of Fe-3wt.%Si-1wt.%Al Electrical Steels in Pure Water Saturated with CO_2_

**DOI:** 10.3390/ma14175084

**Published:** 2021-09-05

**Authors:** Gaetano Palumbo, Dawid Dunikowski, Roma Wirecka, Tomasz Mazur, Urszula Lelek-Borkowska, Kinga Wawer, Jacek Banaś

**Affiliations:** 1 Faculty of Foundry Engineering, Department of Chemistry and Corrosion of Metals, AGH University of Science and Technology, Mickiewicza St. 30, 30-059 Krakow, Poland; dundaw@agh.edu.pl (D.D.); lelek@agh.edu.pl (U.L.-B.); jbs@agh.edu.pl (J.B.); 2 Academic Centre for Materials and Nanotechnology, AGH University of Science and Technology, Mickiewicza St. 30, 30-059 Kraków, Poland; roma.wirecka@gmail.com (R.W.); tmazur@agh.edu.pl (T.M.); 3 Department of Condensed Matter Physics, Faculty of Physics and Applied Computer Science, AGH University of Science and Technology, Mickiewicza St. 30, 30-059 Krakow, Poland; 4 Łukasiewicz Research Network–Institute of Aviation, Al. Krakowska 110/114, 02-256 Warsaw, Poland; Kinga.Wawer@ilot.lukasiewicz.gov.pl

**Keywords:** silicon steel, electrical steel, grain size, electrochemical corrosion, carbon dioxide, AM-KPFM Volta potential measurements

## Abstract

The corrosion behavior of two silicon steels with the same chemical composition but different grains sizes (i.e., average grain area of 115.6 and 4265.9 µm^2^) was investigated by metallographic microscope, gravimetric, electrochemical and surface analysis techniques. The gravimetric and electrochemical results showed that the corrosion rate increased with decreasing the grain size. The scanning electron microscopy/energy dispersive x-ray spectroscopy and X-ray photoelectron spectroscopyanalyses revealed formation of a more homogeneous and compact corrosion product layer on the coarse-grained steel compared to fine-grained material. The Volta potential analysis, carried out on both steels, revealed formation of micro-galvanic sites at the grain boundaries and triple junctions. The results indicated that the decrease in corrosion resistance in the fine-grained steel could be attributed to the higher density of grain boundaries (e.g., a higher number of active sites and defects) brought by the refinement. The higher density of active sites at grain boundaries promote the metal dissolution of the and decreased the stability of the corrosion product layerformed on the metal surface.

## 1. Introduction

Electrical steels, also referred to as silicon steels (i.e., Si is the major additive element), are used as soft magnetic materials for construction of stators and rotors due to their magnetic properties and low cost [1,2]. The magnetic properties of the electrical steel are influenced by different parameters, such as sheet thickness, chemical composition and microstructure, and particularly by the grain size. Previous studies reported that large grain sizes are desired to improve the soft magnetic properties of the electrical steel [3,4]. Lee et al. [4] analyzed the magnetic properties of electrical steel as function of the grain size and found that samples with finer grains exhibited approximately 15% higher core loss W with little effect on the magnetic flux density B, compared to samples with larger grains. It should be noted that electrical steels with a low core loss and high magnetic flux density are preferable for electrical machinery cores from the magnetic properties point of view [4].

Stators and rotors are composed of hundreds of thin electrical steel sheets and used in the cores of electromagnetic devices [2]. Although this equipment is designed to avoid the introduction of any liquids, they usually operate in severe working environments, such as high pressure, high temperature, and presence of aggressive gases (e.g., CO_2_, H_2_S), which can easily compromise their mechanical integrity. With time, the steam condenses into droplets of liquid inside this equipment. The CO_2_ corrosion in the oil and gas industry is one of the greatest challenges [5,6]. The gaseous CO_2_ dissolves in the condensed water, forming carbonic acid, which successively dissociates into bicarbonate and carbonate anions [5,6]. The combination of liquid water and CO_2_ creates aggressive conditions, which may lead to severe corrosion attack, leading to their performance degradation and hence, compromising the functionality of the plant over time. The grain size plays an important role in the design of electrical steel. From the magnetic properties point of view, large grains are more benefical. Grain size has also a strong effect on the mechanical and corrosion properties of the steel [7,8,9,10,11]. The relationship between the grain size and mechanical properties of the steel is well defined by the Hall-Petch relationship. However, the correlation between the grain size and its corrosion behavior is still an open field for investigation. Onyeji et al. [8] studied the corrosion behavior of two X65 steels with the same chemical composition but different grain sizes in aerated and deaerated brine solutions. The authors reported that the steel with coarser grains showed a higher corrosion resistance in both solutions. Li et al. [12] observed that the corrosion resistance of nanocrystallized low-carbon steels in 0.05 M H_2_SO_4_ + 0.05 M Na_2_SO_4_ aqueous solution increased with decreasing the grain size. Palumbo et al. [13] observed that an increase in grain refinement leads to an increase in the volume fraction of intercrystalline areas such as grain boundaries and triple junctions. Many authors argued that the grain boundaries and triple junctions have higher energies compared to the bulk and, as such, are more chemically active with respect to the adjacent matrix [7,8,11,12,14,15,16,17]. Therefore, the grain refinement enhances the reactivity of the surface, which may cause a preferential dissolution of the grains [7,8,11,12,14,15,16,18]. However, it is worth mentioning that there is not an unanimous consensus regarding the effect of the grain size on the corrosion resistance of ferrous alloys. Some studies showed that the environment plays a crucial role. Wang et al. [7] reported that the grain refinement decreased the corrosion resistance of the low alloy steel in a 3.5 wt.% NaCl solution, but the same steel showed an improvement in corrosion resistance in a 0.1 M NaHCO_3_ solution. Similar behavior was observed by Zeiger et al. [16]. The authors found that the grain refinement led to a decrease in the corrosion resistance of the steel in a Na_2_SO_4_ solution with pH = 1, but the corrosion resistance increased in a Na_2_SO_4_ solution with pH = 6. The little consensus reported in the literature is related to the difficulty of isolating the effect of the grain size from other microstructural changes introduced during the grain refinement processes such as, for example, rolling or plastic deformation. Consequently, a case-by-case study is needed to understand the corrosion effect of the grain size of a given metal in a given environment.

The objective of this work is to study the corrosion behavior of two electrical steel sheets with similar chemical composition, but different grain sizes in pure water saturated with CO_2_. To this end, the study was carried out using weight loss and electrochemical measurements. The scanning electron microscopy-energy dispersive x-ray spectroscopy (SEM-EDS) and x-ray photoelectron spectroscopy (XPS) analyses were employed to characterize the corrosion product layer and to support the gravimetric and electrochemical results. Moreover, to highlight the micro-galvanic activities occurring at the grain boundaries and triple junctions on the metal surface, Volta potential measurements were performed.

## 2. Experimental Procedures

### 2.1. Materials

The study was performed on two types of silicon steels labeled 200 and 300. The samples were supplied by Łukasiewicz Research Network according to IEC 60404 8 5 standard. Both samples in as-received conditions were covered with a phosphate protective coating. Each time prior to a test, the coating was removed by grinding the surface with SiC abrasive paper up to 1200 grit and finishing the surface with levigated alumina, cleaned ultrasonically in absolute ethanol and dried before immersion in the tested solution.

The coating-free sample surface was analyzed on a spark spectrometer to identify the chemical composition of samples (Table 1).

### 2.2. Metallographic Analysis

The metallographic analysis was carried out by etching the samples with a nital solution (e.g., 4% HNO_3_ solution) and then degreased with absolute ethanol and dried. Grain size measurements were performed according to the ASTM E112 [19] method using a LEICA DM4000 M LED microscope (Leica Microsystems, Wetzlar, Germany).

### 2.3. Gravimetric Measurement

Gravimetric measurements were carried out by immersing the samples for 24 h in pure water saturated with carbon dioxide at 25 °C. The solution was thermostated in a water bath. After the immersion time had elapsed, the specimens were removed and ultrasonically washed in ethanol, dried, and reweighed. The weight loss was determined using an analytical balance with the accuracy of ±0.1 mg. In each case, the experiment was conducted three times. The corrosion rate (*CR*) in mm·y^−1^ was obtained from the following equation [6]:

(1) $${CR\;(\mathrm{mm}\;y}^{-1})=\;\frac{87.6\;\Delta m}{d\;A\;t}$$

where Δ*m* is the weight loss, *A* is the surface of the sample (cm^2^), *d* is the density (7.87 g cm^−3^), and *t* is the immersion time (h).

### 2.4. Electrochemical Measurement

The electrochemical measurements were carried out with a Gamry 600 potentiostat (Gamry Instruments, Warminster, PA, USA). The electrochemical cell consisted of a working electrode, a saturated calomel reference electrode (SCE) and a platinum counter electrode. The experiments were performed at 25 °C in pure water saturated with carbon dioxide. The electrolyte conductivity was 190 µS cm^−1^ and the pH was 4.12. The tests were carried out using electrochemical impedance spectroscopy (EIS) and potentiodynamic polarization (PDP). The EIS measurements were performed at an amplitude of 40 mV in the frequency range from 1 kHz to 10 mHz. Both measurements were carried out at intervals of 3, 6, 12, 18, and 24 h. The PDP measurements were performed by sweeping the potential from −1.0 to −0.4 V vs. SCE with a scan rate of 1 mV s^−1^ after holding the specimen at open circuit potential for 24 h in the tested solutions. The corrosion rate (*CR*) was calculated according to the ASTM G102 [20] using the following equation:

(2) $${CR\;(\mathrm{mm}\;y}^{-1})=\frac{3.27\text{×}10^{-3}\;i_{\mathrm{corr}}\;E_{W}}{d}$$

where *i*_corr_ is the corrosion current density, *E*_w_ is the equivalent weight of the metal, and *d* is the density of the metal. 3.27 × 10^−3^ is the conversion factor.

### 2.5. Surface Analysis

The morphological analyses were carried out on specimens exposed in the tested solution for 24 h, rinsed with deionized water and dried. The surface analysis was performed using SEM-EDS and XPS. The SEM-EDS investigation was performed using a JEOL scanning electron microscope aquiped with a IXRF EDS detector (JEOL, Inc., Peabody, MA, USA). The XPS analysis was performed with a PHI 5000 VersaProbe II spectrometer (ULVAC-PHI, Inc., Kanagawa, Japan) with an Al Kα monochromatic X-ray beam as described elsewhere [6].

The amplitude modulation Kelvin probe force microscopy (AM-KPFM) Volta potential (ΔΨ) analysis was carried out with a Dimension Icon XR (Bruker, Santa Barbara, CA, USA) working in the tapping mode, using platinum-iridium coated, electrically conductive SCM-PIT-V2 probes with cantilevers with a nominal spring constant of 3.0 N/m. AM-KPFM mode was used with 500 mV bias voltage and 100 nm lift height.

## 3. Results and Discussion

### 3.1. Metallographic Measurement

**Figure 1:**
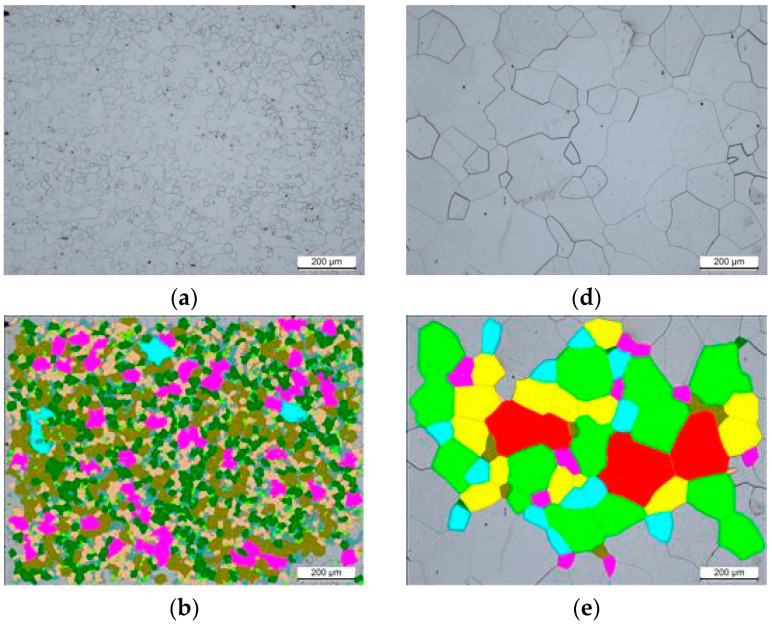

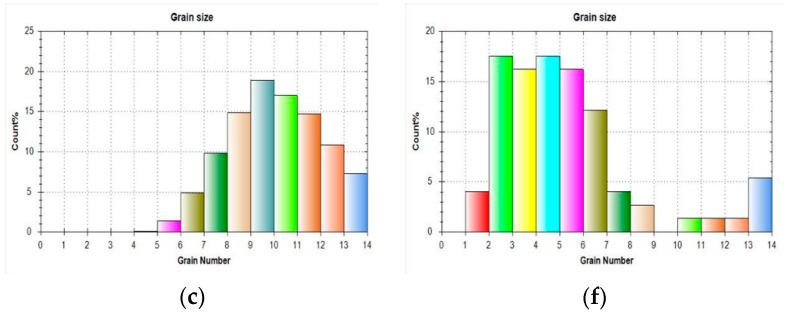
Microstructure and the histogram showing the grain size number distribution of 200 (**a**–**c**) and 300 steels (**d**–**f**).

Figure 1 shows the microstructures and the grain size distribution of the tested steels, with the microstructure parameters summarized in Table 2. It is clear from the data that both samples show a similar chemical composition, with very little variation between different elements, but different microstructures. The 300 steel shows a coarse-grained microstructure with an average grain size of *circa* 4265.9 µm^2^, whereas the 200 steel shows a fine-grained microstructure with an average grain size of *circa* 115.6 µm^2^.

### 3.2. Gravimetric Measurements

Table 3 shows the corrosion rates of the samples obtained from the weight loss measurements after 24 h of immersion in the tested solution. It follows from the data that the *CR* of the sample with fine grains is approximately two times higher compared to the sample with coarse grains. The analysis of the chemical composition (Table 1) shows that both specimens have a similar composition, with very little variation in concentration of the alloying elements. It can be inferred that the small differences observed in microalloying do not affect the corrosion rate of the metal and that the difference in the corrosion rate is therefore related to the microstructure. Similar results were also reported in [8,11,12,14,15,16,18]. Onyeji et al. [8] reported that the corrosion behavior of two X65 steels with identical chemical composition but different microstructures varied. The authors observed that the coarse-grained steel showed higher corrosion resistance compared to the steel with a fine grained microstructure. The authors suggested that this behavior was attributed to the enhanced reactivity of the surface after the grain refinement, which causes a preferential dissolution of the grains.

### 3.3. Electrochemical Measurements

**Figure 2:**
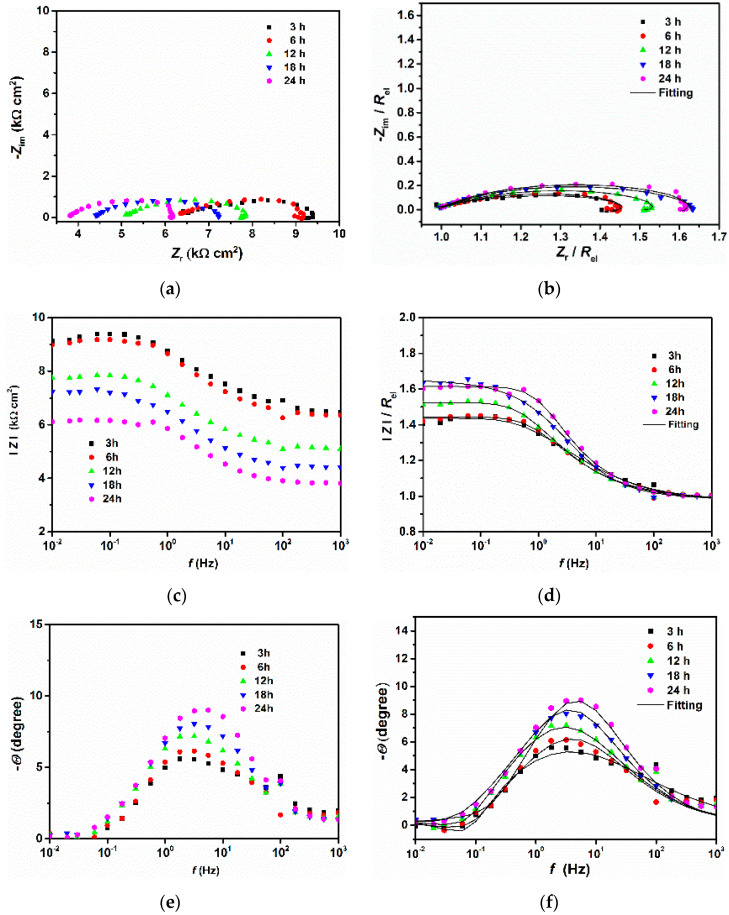
EIS plots obtained after different immersion times for the 200 steel before (**a**,**c**,**e**) and after (**b**,**d**,**f**) the IR drop correction.

**Figure 3:**
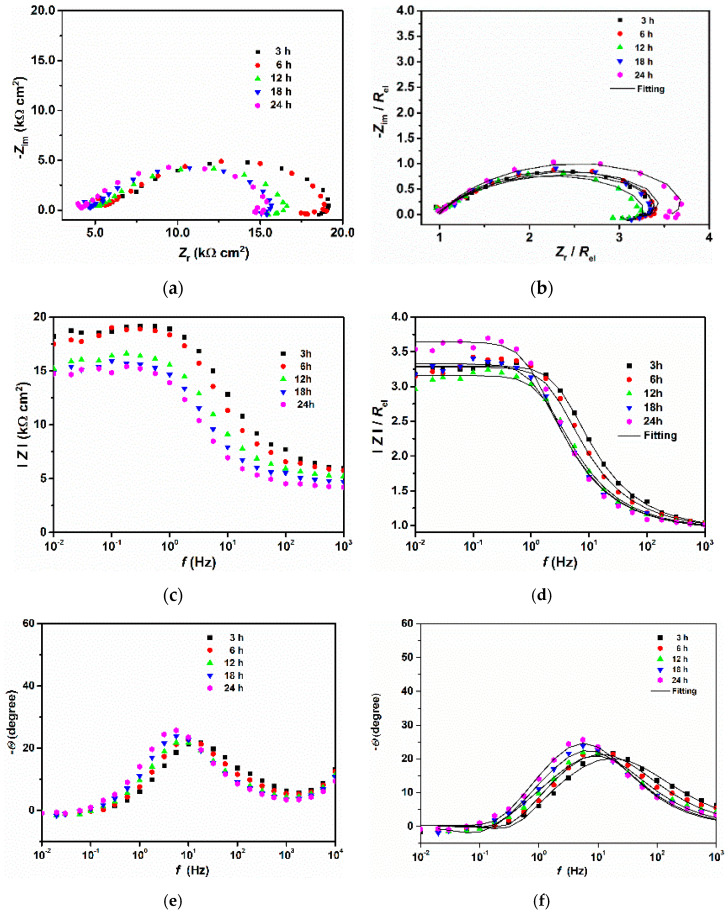
EIS plots obtained after different immersion times for the 300 steel before (**a**–**c**) and after (**d**–**f**) the IR drop correction.

**Figure 4:**
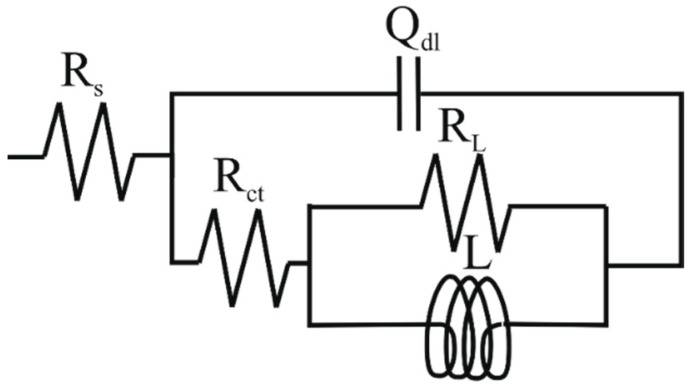
Equivalent circuit used to fit the EIS plots. Here, *R*_s_ is the electrolyte resistance, *Q_d_*_l_ is the constant phase element representing the double charge layer capacitance and *R*_ct_ is the charge transfer resistance. *L* and *R*_L_ represent the inductance and inductance resistance, respectively.

Figure 2 and Figure 3 show the EIS plots carried out under different immersion times at open circuit potential for 200 and 300 steels, respectively. As can be seen from the Nyquist and Bode plots (Figure 2a,c and Figure 3a,c), the conductivity of the solution was very low. This result is understandable since the tested electrolyte consisted of pure water saturated with CO_2_ with a conductivity of approximately 190 μS cm^−1^. However, as time increased, the conductivity of the solution increased, likely due to the release of ions into the bulk solution from the metal surface. To compare the corrosion behavior of both samples, the IR drop was manually compensated. The corrected plots (Figure 2b,d,f, and Figure 3b,d,f) were then fitted with the equivalent circuit displayed in Figure 4 Due to the imperfection of the metal surface, the double-layer capacitance (*C*_dl_) was simulated using a constant phase element (CPE) [14]. The impedance of the CPE is described by the following equation [5,21]:

(3) $$Z_{\mathrm{CPE}}=\frac{1}{{Q(j\omega )}^{n}}$$

where *Q* stands for CPE constant, *n* is the exponent, *j* is the imaginary unit, and *ω* is the angular frequency at which *Z* reaches its maximum value.

As can be seen from figures (Figure 2b,d,f and Figure 3b,d,f), the fitted results were similar to those obtained experimentally and the values of *χ*^2^ were very low (Table 4), indicating that the equivalent circuit, employed to simulate the system under investigation, was the most appropriate one. It follows from the Nyquist diagrams (Figure 2c and Figure 3c) that the shape of the curve did not change with the immersion time and exhibited a depressed semicircle in the whole frequency range due to the inherent charge transfer processes controlling the corrosion reactions. An inductive loop is also visible at low frequencies, likely due to the relaxation time of the intermediate adsorbed species.

The CO_2_ gas dissolves in the solution forming carbonic acid, which successively dissociates into bicarbonate and carbonate anions, according to the following reactions:

(4) $${\mathrm{CO}}_{2}{+H}_{2}O_{\left(l\right)}\;↔{\;H}_{2}{\mathrm{CO}}_{3}$$

(5) $$H_{2}{\mathrm{CO}}_{3}\;↔{\;H}^{+}{+\mathrm{HCO}}_{3}^{-}$$

(6) $${\mathrm{HCO}}_{3}^{-}\;↔{\;H}^{+}{+2\mathrm{CO}}_{3}^{2-}$$

In the presence of CO_2_, the process is controlled by the three cathodic reactions [6,22,23]:

(7) $${2H}_{2}{\mathrm{CO}}_{3}{+2e}^{-}\;\to {\;H}_{2}{+2\mathrm{HCO}}_{3}^{-}$$

(8) $${2\mathrm{HCO}}_{3}^{-}{+2e}^{-}\;\to {\;H}_{2}{+2\mathrm{CO}}_{3}^{2-}$$

(9) $${2H}^{+}{+2e}^{-}\;\to {\;H}_{2}\;$$

The anodic reaction in a CO_2_-saturated solution can be summarized by the multi-step dissolution of carbon steel [24]:

(10) $${\mathrm{Fe}+H}_{2}O_{\;}\to {\;(\mathrm{FeOH})}_{\mathrm{ads}}{+H}^{+}{+e}^{-}$$

(11) $${\left(\mathrm{FeOH}\right)}_{\mathrm{ads}}\;\to {\;\mathrm{FeOH}}^{+}{+e}^{-}\;$$

(12) $${\mathrm{FeOH}}^{+}{+H}^{+}\;\to {\;\mathrm{Fe}}^{2+}{+H}_{2}O_{\;}\;$$

The inductive loop, observed at low frequencies, is likely due to the adsorption of (FeOH)_ads_ on the metal surface [24].

**Figure 5:**
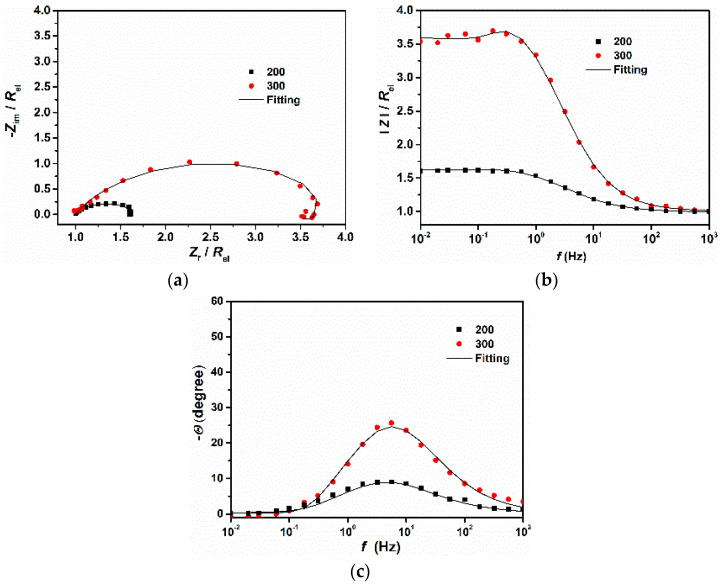
EIS plots comparing the two steels after 24 h of immersion and after the IR drop correction in the tested solution. (**a**) Nyquist, (**b**) Bode and (**c**) Phase angle.

After 24 h of immersion the 300 steel showed a higher capacitive semicircle compared to the 200 steel (Figure 5) The EIS findings are in agreement with the gravimetric results. Since the corrosion resistance of a given metal is a function of the size of the capacitive loop, it follows from the figure that the 300 steel, with a wider Nyquist curve capacitive loop, shows a higher corrosion resistance than the 200 steel. The SEM-EDS and XPS analyses (Section 3.4) indicate that the higher corrosion resistance of the 300 steel was likely ascribed to formation of a more stable and/or compact protective layer comprised of Al_2_O_3,_ SiO_2_, Fe_2_O_3_ and traces of FeCO3. Since both materials had a similar chemical composition this behavior can be ascribed to different microstructure. Di Schino et al. also observed a similar result [9,10]. They studied the effects of the grain size on the corrosion behavior of refined austenitic stainless steel in a 5% H_2_SO_4_ boiling solution after 10 h of immersion. The authors reported that the corrosion rate decreased with increasing the grain size. They suggested that an increase of the grain boundary surface area due to grain refining, caused the passive film to become less stable, due to the defects concentrated in the grain boundaries. In their study, the layer formed on the coarse-grained steel was more stable and could thus provide more protection to the steel substrate by a blocking effect, thereby reducing the diffusion of the aggressive substances from the bulk solution to the metal surface.

**Figure 6:**
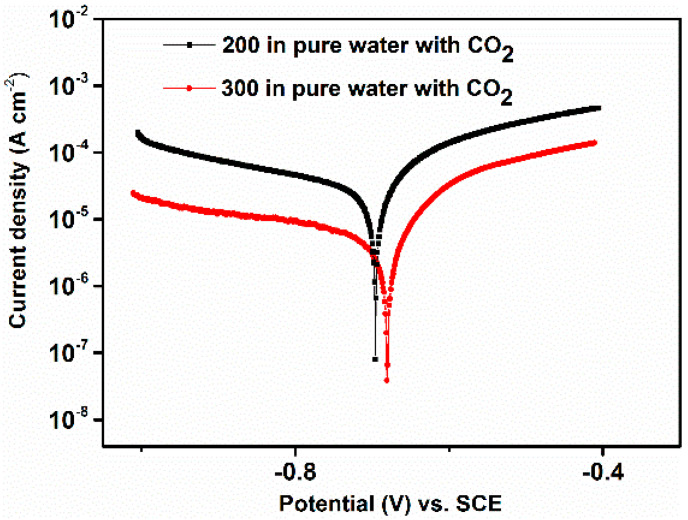
Potentiodynamic polarization curves obtained after 24 h of immersion in the tested solution.

Figure 6 and Table 5 show the potentiodynamic polarization measurements and corrosion kinetic parameters observed after 24 h of immersion in the tested solution, respectively. It is evident from the data that the corrosion current density of the steel with coarse grains was significantly lower compared to steel with fine grains. The corrosion rate of the metal with coarse grains was found to be 0.15 mm y^−1^ against 0.28 mm y^−1^ for the metal with fine grains, in agreement with the results observed with the gravimetric measurements. Furthermore, both the anodic and cathodic branches of the polarization curves were shifted towards the lower current densities for the steel with coarser grains. Li et al. [12] also reported a similar result. The authors suggested that the higher anodic current density observed for the steel with finer grains was related to the high energies that the atoms have at the grain boundaries. These atoms are the first to take part in the reaction. An increase in grain refinement leads to an increase in the volume fraction of intercrystalline areas such as grain boundaries and triple junctions [13]. Therefore, as the volume fraction of the grain boundary increases, the amount of the active atoms on the steel surface also increases, which in turn leads to an increase in the anodic current density. The result suggests that the stable layer, formed on the coarse grain size metal surface, hinders both the rate of the cathodic reaction (Equations (7)–(9)) and anodic dissolution (Equations (10)–(12)), by either covering part of the metal surface or blocking the active corrosion sites on the steel surface [5,6].

**Figure 7:**
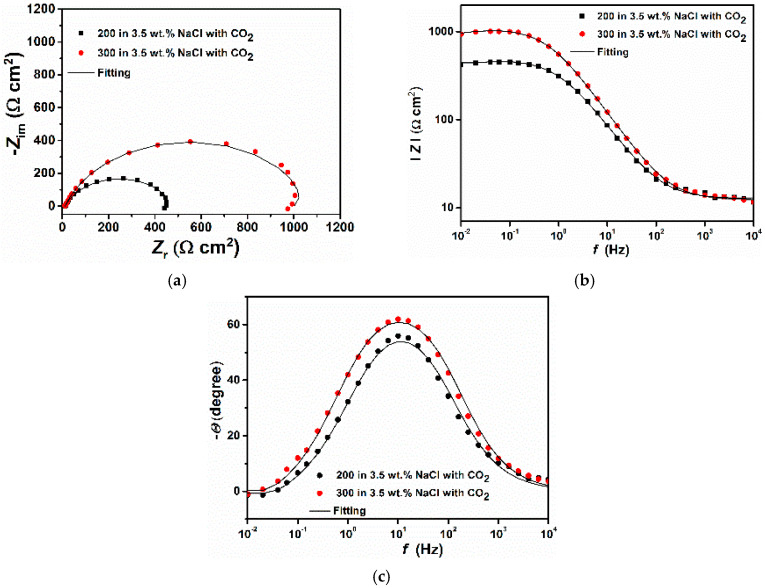
EIS plots comparing the two different samples after 24 h of immersion in a 3.5 wt.% NaCl saturated with CO_2_. (**a**) Nyquist, (**b**) Bode, and (**c**) Phase angle.

**Figure 8:**
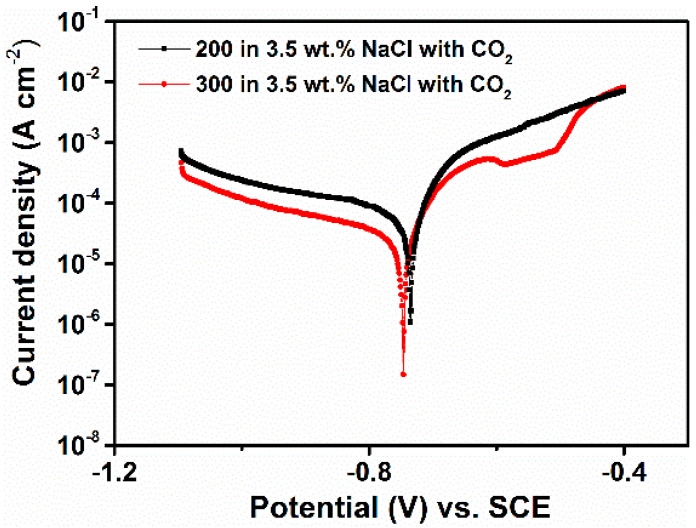
Potentiodynamic polarization curves obtained after 24 h of immersion in a 3.5 wt.% NaCl aqueous solution saturated with CO_2_.

To confirm the correctness of the results obtained in pure water, the electrochemical experiments were also carried out in a 3.5 wt.% NaCl aqueous solution saturated with CO_2_. The EIS and PDP results are displayed in Figure 7 and Figure 8 and Table 6 and Table 7, respectively. The results are in agreement with ones observed in pure water saturated with CO_2_, which shows that the coarse-grained steel still displays a better corrosion resistance compared to the fine grained steel. In particular, it can be seen from the potentiodynamic experiments (Figure 8) that the 300 steel exhibits a pseudo-passive region, only, extends to a small range of potential (i.e., −612 to −586 mV vs. SCE). This result confirms that the coarse-grained steel tends to form a more stable corrosion product layer on the surface, which led to an increase in its corrosion resistance.

### 3.4. Morphological Analysis

**Figure 9:**
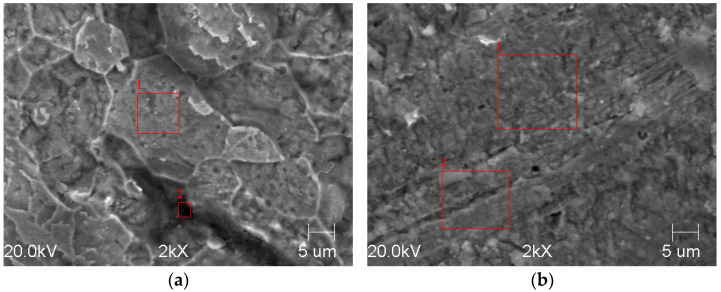
SEM analysis on the sample (**a**) 200 and (**b**) 300, after 24 h of immersion. (The red square area corresponds to the area of the EDS analysis).

Figure 9 shows the surface morphology after 24 h of immersion in pure water saturated with CO_2_. It is clear from figures that the surface morphology of the tested samples differs significantly. The severity of the corrosion process revealed the microstructure of the 200 steel (Figure 9a). By contrast, the surface of the 300 steel appears much smoother. The EDS analysis listed in Table 8 confirmed formation of a protective corrosion product layer, mainly composed of aluminum, oxygen, and other alloying elements. However, the data shows that the surface of the 300 steel was covered by a more homogeneous layer with the concentration of the abovementioned elements uniformly distributed over the entire surface. On the other hand, the surface of the 200 steel shows the presence of darker areas (e.g., red square 2 in Figure 9a), where the concentration of Al and O was higher compared to lighter areas (e.g., red square 1 from Figure 9a) and much similar to that found on the surface of the 300 steel. These results confirmed the observations reported in this study and are in agreement with the literature [9,10,11,12,13,14,15,16]. The results suggest that the better corrosion resistance performance observed of the coarse-grained steel was ascribed to its ability to form a more homogeneous and stable layer over the entire surface, which shields the material from the aggressive solution. Moreover, the morphological analysis also shows the presence of carbon on both surfaces. The presence of carbon was likely due to the precipitation of FeCO_3_. Iron carbonate can form when the concentration of Fe^2+^ and ${\mathrm{CO}}_{3}^{2-}$ ions exceeds its solubility product (i.e., super-saturation):

(13) $${\mathrm{Fe}}^{2+}{+\mathrm{CO}}_{3}^{2-}\;\to {\;\mathrm{FeCO}}_{3}\;$$

**Figure 10:**
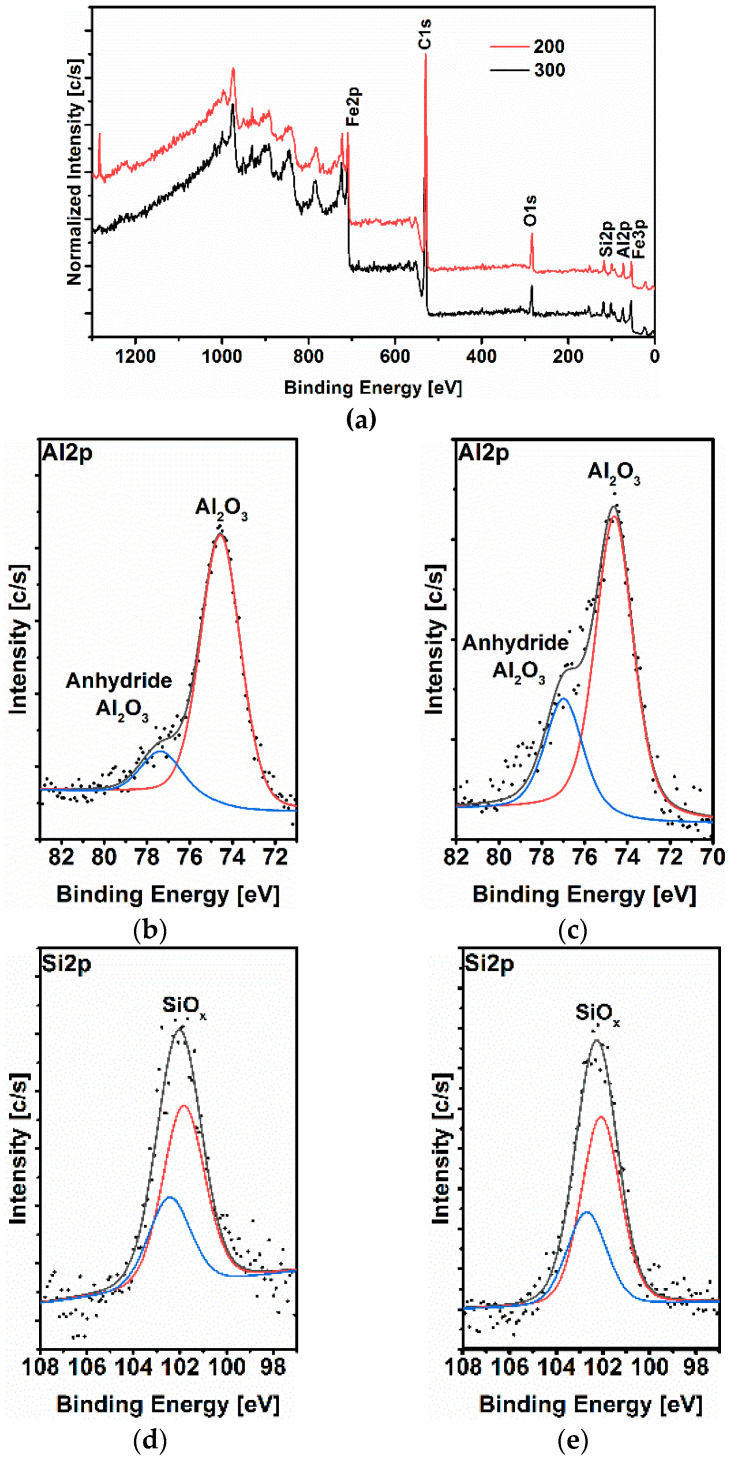

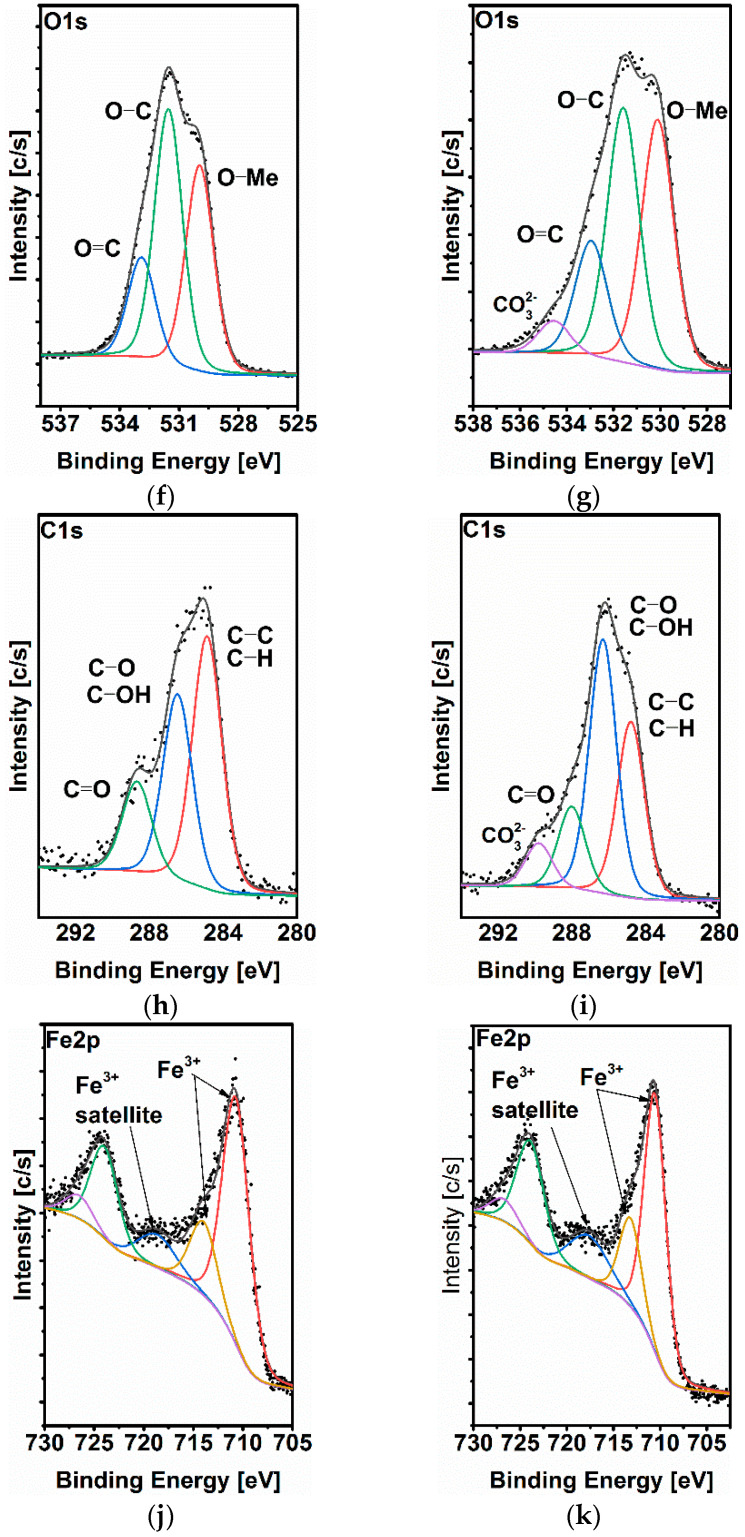
XPS analysis carried out after 24 h of immersion. Survey (**a**), 200 steel (**b**,**d**,**f**,**h**,**j**) and 300 steel (**c**,**e**,**g**,**i**,**k**).

The precipitation of FeCO_3_ depends not only on the concentration of Fe^2+^ and ${\mathrm{CO}}_{3}^{2-}$ ions, but is also affected by other factors such as temperature, CO_2_ partial pressure, and pH. Among the abovementioned factors, the pH of the solution can be regarded as one of the most influential factors [25]. Dugstad [25] reported that an increase in pH of the solution significantly reduced the concentration of Fe^2+^ ions required to exceed the FeCO_3_ solubility product and therefore promoteing its precipitation. In this study, the pH increased as the immersion time increased, going from 4.12, at the beginning of the experiment, to 5.61 after 24 h of immersion. As such, making the precipitation of FeCO_3_ more probable. Moreover, Dugstad [25] also reported that the concentration of Fe^2+^ ions is higher at the surface/solution interface compared to the bulk solution. Consequently, the concentration of Fe^2+^ ions required to promote the formation of FeCO_3_ on the metal surface is lower and thus, increasing the likelihood of having FeCO_3_ on surface. However, only small traces of FeCO_3_ could be found as confirmed by the XPS analysis (Figure 10). Previous studies reported that FeCO_3_ begins to decompose at temperatures below 100 °C according to the following reaction [6,23,26]:

(14) $${\mathrm{FeCO}}_{3}\;\to {\;\mathrm{FeO}+\mathrm{CO}}_{2}$$

In the presence of CO_2_ or water vapor, FeO transforms into Fe_3_O_4_ [23,26].

(15) $${3\mathrm{FeO}+\mathrm{CO}}_{2}\;\to {\;\mathrm{Fe}}_{3}O_{4}+\mathrm{CO}$$

(16) $$3\mathrm{FeO}+\;H_{2}O\;\to {\;\mathrm{Fe}}_{3}O_{4}+\;H_{2}$$

In the presence of oxygen, FeO and Fe_3_O_4_ transform into Fe_2_O_3_ [23,26].

(17) $$4\mathrm{FeO}+\;O_{2}\;\to {\;2\mathrm{Fe}}_{2}O_{3}$$

(18) $$\mathrm{In}\;\mathrm{the}\;\mathrm{air}:\;4{\mathrm{Fe}}_{3}O_{4}{+O}_{2}\;\to {\;6\mathrm{Fe}}_{2}O_{3}$$

The XPS was employed to characterize the composition of the thin corrosion layer formed after 24 h of immersion at 25 °C (Figure 10). The high-resolution spectra of aluminum (Al2p), silicon (Si2p), oxygen (O1s), carbon (C1s), and iron (Fe2p) are presented in Figure 10 and the binding energies and the corresponding quantification (%) of each peak are presented in Table 9. The deconvolution of the Al2p spectrum (Figure 10b,c) shows two peaks located at 74.7 and ~77.5 eV that can be attributed to Al_2_O_3_ and anhydrous Al_2_O_3_, respectively [27]. The high-resolution XPS spectrum of Si2p (Figure 10d,e) shows two main peaks at around 102 and 102.5 eV corresponding to Si-O/Si-O-C bond (Silicon oxide/Silicon oxycarbide). The silicon oxycarbide phase might have been formed on the surface as part of the protective layer when the material was exposed to the atmosphere. The O1s spectrum (Figure 10f,g) is fitted into three distinct peaks namely, 530, 531.6, and 533 eV. The peak observed at 530 eV was ascribed to O^2−^ and could be related to oxygen atoms bonded to the metal, (i.e., Al_2_O_3_, Fe_2_O_3_, and SiO_2_ oxides) [6], whereas the peaks at 531.6 and 533 eV are associated with single bonded and double-bonded oxygen in FeCO_3_ [6,28]. A fourth peak was observed at 354.6 eV for the 300 steel and can be attributed to the COO^−^ of FeCO_3._ The C1s spectrum (Figure 10h,i) corroborated with the data observed for the O1s spectrum, which shows three peaks at 284.8, ~286.3, and ~288.5 eV. The 284.6 eV peak may correspond to secondary carbon [28], whereas the peaks at ~286.3 are ~288.5 eV are attributed to the C–O and C=O bonds of FeCO_3_ [6]. As in the case of the O1S spectrum, for the 300 steel, a fourth peak was observed at 289.8 eV, which is characteristic of FeCO_3_ [29]. The deconvoluted Fe2p_3/2_ peaks (Figure 10j,k) could be attributed to α-Fe_2_O_3_ or/and γ- Fe_2_O_3_ oxides [6,30], likely due to the partial decomposition of iron carbonate (i.e., Equations (14)–(18)).

The XPS analysis is in agreement with the EDS analysis (Table 8), indicating that the corrosion products formed on both samples were mainly composed of Al_2_O_3_ and SiO_2_, with traces of FeCO_3_/Fe_2_O_3_.

**Figure 11:**
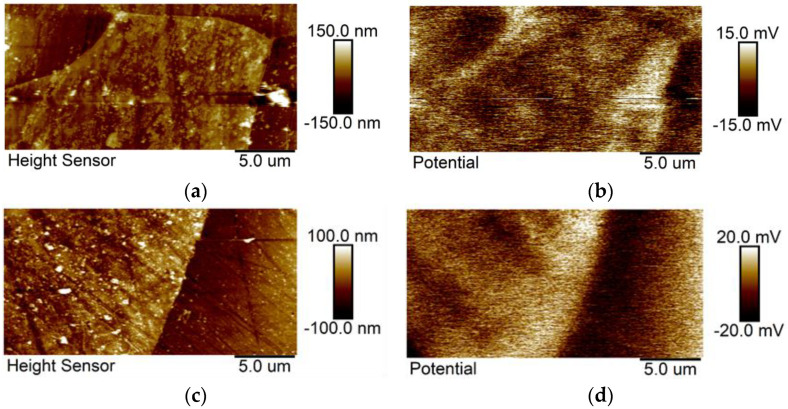
AM-KPFM analysis of the steels with the topography and the corresponding Volta potential map, respectively. Steel 200 (**a**,**b**); 300 (**c**,**d**).

To study the micro-galvanic activities occurring at the grain boundaries and triple junction on the metal surface, AM-KPFM measurements were carried out. AM-KPFM is a powerful technique for assessing the Volta potential (Δ*Ψ*) of a metal surface. The Δ*Ψ* is a characteristic property of the metal surface and can provide an insight into the local electrochemical activities on the metal surface [31]. Figure 11 the topography and the corresponding Volta potential maps of the two tested steels. The Volta potential maps show darker color representing the anodic regions, whereas the lighter color representing cathodic regions. The Δ*Ψ* mapping clearly shows potential differences at grain boundaries and triple junctions, indicating higher electrochemical activity in these regions. The grain boundaries and triple junctions are characterized in the maps as lighter zones, thus representing the cathodic areas and displaying a relative Δ*Ψ* difference of *circa* +30 mV with respect to the adjacent matrix. The results confirmed that these regions are more active compared to the adjacent matrix, which makes them more susceptible to corrosion attack during the exposure to electrolytes. Therefore, the grain refinement enhances the reactivity of the surface, which could promote a preferential dissolution of the grains [7,8,11,12,14,15,16,18].

## 4. Conclusions

The effect of the grain size on the corrosion behavior of two electric steels in pure water saturated by CO_2_ can be summarized as follows:

- The gravimetric results indicated that the grain refinement decreases the corrosion resistance of the steel.
- The EIS measurements showed that the coarse-grained sample displayed a higher capacitive loop compared to fine-grained steel. This result is related to the formation of a thicker and more stable protective corrosion product layer.
- The potentiodynamic measurements showed that the corrosion current density of the coarse-grained steel was much smaller compared fine-grained steel. Both the anodic and cathodic current densities were found to be lower for the coarse-grained steel.
- The SEM-EDS and XPS analyses confirmed presence of a thicker and more homogenous protective layer on the coarse-grained steel, consisting mainly of Al_2_O_3_, SiO_2_, and traces of FeCO_3_.
- The Volta potential measurements showed potential differences between the grains and grain boundaries, indicating a higher electrochemical activity in these regions, which would couse a preferential dissolution of grains.

## Figures and Tables

**Table 1:** Chemical composition of the tested samples.

Element	Sample	
wt.%	200	300
C	0.011 ± 0.01	0.007 ± 0.01
Si	2.93 ± 0.17	3.24 ± 0.11
Mn	0.22 ± 0.03	0.18 ± 0.02
P	0.03 ± 0.01	0.026 ± 0.03
S	0.004 ± 0.001	0.003 ± 0.001
Al	0.91 ± 0.13	1.05 ± 0.08
Fe	Bal.	Bal.

**Table 2:** Grain size analysis of the steels.

200			300		
Average grain size number (G)	Average diameter (µm)	Average grain area (µm^2^)	Average grain size number (G)	Average diameter (µm)	Average grain area (µm^2^)
10.1 ± 0.23	10.8 ± 0.87	115.6 ± 20.9	4.9 ± 0.32	65.3 ± 7.49	4265.9 ± 499.7

**Table 3:** Weight loss and corrosion rate of the steels after 24 h of immersion time.

Sample	Weight Loss (mg)	Corrosion Rate (mm y^−1^)
200	3.57 ± 0.25	0.25 ± 0.02
300	0.79 ± 0.04	0.12 ± 0.01

**Table 4:** Electrochemical impedance parameters of the two steels in the tested solution after the IR drop correction.

Sample	Time (h)	*R*_s_ (Ω cm^2^)	*Q*_1_ (mΩ^−1^ s^n^ cm^−2^)	*n*	*R*_ct_ (Ω cm^2^)	*L* (H cm^2^)	*R*_L_ (Ω cm^2^)	*χ*^2^ (10^−4^)
200	3	0.96	0.48 ± 0.06	0.57 ± 0.02	0.47 ± 0.03	0.27 ± 0.03	0.14 ± 0.011	1.05
	6	0.98	0.38 ± 0.03	0.59 ± 0.04	0.45 ± 0.05	0.15 ± 0.02	0.01 ± 0.005	0.99
	12	0.98	0.36 ± 0.03	0.58 ± 0.05	0.54 ± 0.06	0.19 ± 0.04	0.01 ± 0.003	0.38
	18	0.97	0.26 ± 0.02	0.63 ± 0.03	0.65 ± 0.03	0.12 ± 0.02	0.04 ± 0.001	0.46
	24	0.99	0.19 ± 0.01	0.67 ± 0.05	0.64 ± 0.03	0.07 ± 0.01	0.07 ± 0.002	1.6
300	3	0.95	0.04 ± 0.001	0.61 ± 0.04	2.32 ± 0.16	0.24 ± 0.01	0.70 ± 0.06	2.02
	6	0.98	0.04 ± 0.001	0.65 ± 0.03	2.35 ± 0.21	0.25 ± 0.02	0.69 ± 0.02	2.06
	12	0.89	0.04 ± 0.003	0.69 ± 0.04	2.03 ± 0.22	0.64 ± 0.01	0.49 ± 0.02	1.99
	18	0.98	0.05 ± 0.005	0.71 ± 0.03	2.26 ± 0.15	0.36 ± 0.02	0.52 ± 0.03	1.41
	24	0.97	0.05 ± 0.003	0.72 ± 0.02	2.61 ± 0.28	0.36 ± 0.01	0.51 ± 0.05	0.94

**Table 5:** Potentiodynamic polarization parameters in the tested solution obtained after 24 h of immersion.

Sample	*E*_corr_ (V vs. SCE)	−*β*_c_ (V dec^−1^)	*i*_corr_ (μA cm^−2^)	*CR* (mm y^−1^)
200	−0.696 ± 0.015	0.433 ± 0.087	24.44 ± 1.87	0.28 ± 0.02
300	−0.680 ± 0.011	0.588 ± 0.051	12.98 ± 2.57	0.15 ± 0.03

**Table 6:** Electrochemical impedance parameters of the steels after 24 h of immersion in a 3.5 wt.% NaCl aqueous solution saturated with CO_2_.

Sample	Time (h)	*R*_s_ (Ω cm^2^)	*Q*_1_ (mΩ^−1^ s^n^ cm^−2^)	*n*	*R*_ct_ (Ω cm^2^)	*L* (H cm^2^)	*R*_L_ (Ω cm^2^)	*χ*^2^ (10^−4^)
200	24	10.04	0.46 ± 0.03	0.78 ± 0.03	421.10 ± 28.53	50.11 ± 9.07	49.22 ± 8.59	8.27
300	24	10.26	0.30 ± 0.06	0.80 ± 0.05	968.30 ± 40.89	60.10 ± 10.03	31.81 ± 6.89	5.69

**Table 7:** Potentiodynamic polarization parameters obtained after 24 h of immersion in a 3.5 wt.% NaCl aqueous solution saturated with CO_2_.

Sample	*E*_corr_ (V vs. SCE)	−*β*_c_ (V dec^−1^)	*i*_corr_ (μA cm^−2^)	*CR* (mm y^−1^)
200	−0.735 ± 0.019	0.517 ± 0.044	70.42 ± 2.34	0.82 ± 0.03
300	−0.746 ± 0.016	0.471 ± 0.067	29.43 ± 1.37	0.34 ± 0.02

**Table 8:** EDS analysis of the tested samples.

Element (wt.%)	200		300	
	1	2	1	2
C	11.97 ± 2.95	23.04 ± 2.17	21.78 ± 0.08	20.83 ± 1.35
O	7.11 ± 1.62	14.75 ± 1.04	10.71 ± 1.44	14.38 ± 1.03
Al	0.90 ± 0.08	3.54 ± 0.53	4.96 ± 0.55	7.10 ± 0.47
Si	2.60 ± 0.14	3.39 ± 0.30	3.19 ± 0.10	3.50 ± 0.05
P	0.09 ± 0.01	0.24 ± 0.02	0.10 ± 0.01	0.11 ± 0.01
S	0.08 ± 0.03	0.11 ± 0.02	0.11 ± 0.01	0.06 ± 0.01
Mn	0.13 ± 0.03	0.06 ± 0.01	0.07 ± 0.01	0.13 ± 0.01
Fe	77.12 ± 4.58	54.87 ± 1.42	59.12 ± 2.37	50.88 ± 5.55

**Table 9:** XPS analysis of sample surfaces after 24 h of immersion.

Peak Assignment	200		300	
	Binding Energy (eV)	%Area	Binding Energy (eV)	%Area
C1s	-	23.0	-	14.5
C–C, C–H	284.8	47.5	284.8	52.4
C–O, C–OH	286.5	35.2	286.3	24.5
C=O	288.7	17.3	288.5	14.2
${\mathrm{CO}}_{3}^{2-}$	-	-	289.8	8.9
Al2p	-	12.0	-	14.5
Al_2_O_3_	74.7	85.8	74.7	72.6
AlOOH	77.5	14.2	77.3	27.4
Si2p	-	4.6	-	7.2
SiO_x_	101.8	66.7	102.1	66.7
splitting	102.5	33.3	102.7	33.3
O1s	-	53.7	-	54.0
O–Me	530	36.6	530.1	38.2
O–C	531.6	45.3	531.6	38.9
O=C	532.9	18.1	533.0	17.8
${\mathrm{CO}}_{3}^{2-}$	-	-	534.6	5.1
Fe2p	-	5.7	-	8.3
Fe^3+^	710.5	44.5	710.7	51.0
Fe^3+^	713.2	15.2	714.0	15.3
satellite	717.6	13.2	718.6	9.7
-	723.9	22.2	724.0	19.1
-	726.7	4.9	726.5	4.9

## Data Availability

The data presented in this study are available on request from the corresponding author.
